# Non-Medical Use of Novel Synthetic Opioids: A New Challenge to Public Health

**DOI:** 10.3390/ijerph16020177

**Published:** 2019-01-09

**Authors:** Barbara Lovrecic, Mercedes Lovrecic, Branko Gabrovec, Marco Carli, Matteo Pacini, Angelo G. I. Maremmani, Icro Maremmani

**Affiliations:** 1 National Institute of Public Health, 1000 Ljubljana, Slovenia; barbara.lovrecic@nijz.si (B.L.); mercedes.lovrecic@nijz.si (M.L.); branko.gabrovec@nijz.si (B.G.); 2 Centre for Psychiatry and Addiction Medicine, Izola Health Centre, 6310 Izola, Slovenia; 3 Department of Translational Research and New Technologies, University of Pisa, 56100 Pisa, Italy; carlimarco@outlook.it; 4 G. De Lisio Institute of Behavioral Sciences, 56100 Pisa, Italy; paciland@virgilio.it; 5 Department of Psychiatry, North-Western Tuscany Region NHS Local Health Unit, Versilia Zone, 55049 Viareggio, Italy; angelo.maremmani@uslnordovest.toscana.it; 6 Association for the Application of Neuroscientific Knowledge to Social Aims (AU-CNS), Pietrasanta, 55045 Lucca, Italy; 7 Vincent P. Dole Dual Disorder Unit, Santa Chiara University Hospital, University of Pisa, 56100 Pisa, Italy

**Keywords:** new psychoactive substances, new synthetic opioids, illicit fentanyl, Mitragyna speciosa, o-desmethyltramadol, novel fentanyl derivatives, new generation of novel synthetic opioids, harm reduction strategies, comprehensive treatment

## Abstract

*Background*: In the last decade there has been a progressive increase in the use of new psychoactive substances (NPSs) that are not yet under international control. In particular, novel synthetic opioids (NSOs) have reappeared on the recreational drug market in the last few years. As a result, the use of NSOs has increased rapidly. This poses an emerging and demanding challenge to public health. *Aim*: To raise awareness among clinicians and other professionals about NPSs, especially NSOs, to summarize current knowledge about pharmacological properties, forms of NSO on the market, pattern of use, effects and consequences of use. *Methods*: An electronic search was carried out on the Medline/PubMed and Google Scholar databases to find selected search terms. *Results*: Some NPSs are already controlled, while others can be legally sold directly on the drug market (mainly via internet, less so by drug dealers) or be used as precursors for the synthesis of other designer drugs that mimic the psychoactive effects of controlled substances. Potential side-effects of NSOs include miosis, sedation, respiratory depression, hypothermia, inhibition of gastrointestinal propulsion, death (from opioid overdose). *Conclusions*: The severity of the opioid crisis has intensified with the introduction of highly potent NSOs on the drug market. As long as addicts are dying from overdose or similar causes, there is something more constructive to do than waiting for addicts to overdose on heroin at a place located near a remedy, as if to say, within reach of naloxone.

## 1. Introduction

New psychoactive substances (NPSs) are legally defined as new narcotic or psychotropic drugs, in pure form or in a preparation, which are not scheduled under the 1961 Single Convention on Narcotic Drugs or the 1971 Convention on Psychotropic Substances [1], but which may pose a public health threat comparable with classic illicit drugs, substances listed in conventions (Council of the European Union decision 2005/387). According to the United Nations Office on Drugs and Crime (UNODC) [1], the term “new or novel” does not always mean new molecules or inventions, and does not have to refer to the time when a substance was first identified or synthesized (most NPSs were first created many years ago and there is continuous replacement of analogues on the drug market in such a way as to circumvent existing drug control laws and bans); it refers to when that new substance emerged on the global drug market for recreational use (non-medical use), so that its true meaning comes close to “newly misused” [1].

In the last ten years there has been an increase in the use of NPSs that are not yet under international control. Although the widespread availability of NPSs has become a global worldwide phenomenon, the European market currently offers the highest number of NPSs. According to UNODC, counting up to the middle of 2017, 702 of the cumulative global total of 739 different notifications of NPSs to UNODC had been reported by 41 European countries [2]. In the EU in 2016, on average one NPS per week was reported to the EMCCDA, while in 2017 the overall number of NPSs reported was lower than in 2016 [3].

Until 2013 the majority, around two thirds of NPSs, consisted of synthetic cannabinoids and synthetic cathinones, while among opioids only O-desmethyltramadol and kratom were reported [1]. Although the most popular NPSs are still synthetic cannabinoids and synthetic cathinones, novel synthetic opioids (NSOs), in particular fentanyl analogues, stand out as an emerging class among NPSs [2]. In the 2014 to 2016 period the largest percentage increase in NPSs was observed in the group of NSOs (2% of all NPSs in 2014 vs. 4% of all NPSs in 2016) [2]. NSO were reported from North America, Latin America, Canada, Asia, Europe, Africa and elsewhere, and included analogues of fentanyl and various non-fentanyl compounds [2,3]. The phenomenon of inappropriate fentanyl, fentanyl analogues and non-fentanyl compound use comprises multifaceted problems ranging from NPS fentanyl analogues with unlimited availability on the Internet (on the so called deep and dark nets), illicit fentanyl, diversion and misuse of licensed medicines containing fentanyl, fake medicines sold on the illicit market laced with fentanyl and used as heroin substitutes or as adulterants in illicit street drugs [2,4,5,6]. Deaths and other health consequences associated with the use of NSOs were reported from US, Europe, Canada and elsewhere [2].

The purpose of this paper is to gathering and summarizing information, for clinicians and other professionals, about NSOs epidemiology and common characteristics, pointing out their clinical toxicity. This paper further highlights the importance for health and other professionals of considering NSOs as a potential risk to health and, indeed, life itself, critically discussing the current possibility to treat and prevent NPS and NSO misuse.

## 2. Methods

We conducted an electronic search on the PubMed and Google Scholar databases for articles using the following keywords alone or in combination: ‘NPS’, ‘new synthetic psychoactive substances (*n* = 124)’, ‘novel synthetic opioids’ (*n* = 98), ‘novel synthetic illicit opioids’ (*n* = 8), ‘fentanyl’ (*n* = 7009), ‘fentanyl derivatives’ (*n* = 1), ‘illicit fentanyls’ (*n* = 67), ‘U-47700’ (*n* = 30), ‘AH-7921’ (*n* = 30), ‘MT-45’ (*n* = 25), ‘W-18’, (*n* = 8) ‘novel illicit opioid toxicity’ (*n* = 5). Papers were limited to those available in English, covering the period 1 January 2008–31 December 2017, only if they were peer-reviewed and regarding humans (manuscript on animal models were not included) exposed to non-medical use of fentanyl, fentanyl derivates and illicit novel opioids. 397 articles were screened from their abstracts to determine their relevance in the framework of the current review, focusing on reviews, clinical consequences of acute toxicity (e.g., poison center data, case reports) and drug related deaths in humans (with post mortem toxicology confirmation). 112 papers were finally reviewed for this manuscript.

An initial review of the titles and abstracts resulting from these electronic searches was followed by a more detailed assessment of pertinent articles, and an examination of the bibliographies of related reviews to identify other sources. Government sources were also inspected for relevant information. A final search of the electronic databases was conducted in 2018. Review of articles was made by BL and ML. The 2008–2017 period was chosen due to the relevance of the topic.

The recreational use of novel psychoactive substances (NPS) gained worldwide popularity mostly since 2008 (especially after 2008). Over the last ten years there has been a sharp increase in the use of NPS. According to the United Nations Office on Drugs and Crime (UNODC), until 2013 synthetic cannabinoids and synthetic cathinones made up a majority of NPS while, among opioids, only O-desmethyltramadol and kratom were reported. In the period from 2012 to 2016 fourteen fentanyl analogues and three synthetic opioids belonging to other structural groups were reported to the UNODC, in 2014–2017 period novel synthetic opioids increased dramatically.

## 3. Results

### 3.1. Epidemiology

The prevalence of NPS and NSO use is hard to estimate. The dynamic drug market of NPSs, including NSOs, is continuously changing, while NSO users are usually unaware of the NSO content and their exposure to it, or they may know only a brand or street name. In addition, NSOs are not detected by standard toxicology screens, so exposure to NSOs even in cases of toxicity and medical treatment may remain unrecognized and under-reported [7]. Most NPSs and NSOs are, in practice, exempt from international control, so that they may not be seized or reported [2]. On the other hand, in the last few years more evidence has emerged about the harmful effects of using NPSs. Indirect information could be obtained from data on acute clinical toxicity (overdose data) which are limited to retrospective analysis, surveys and case reports, and are mainly reported by poison centers, NSO-related mortality data and data about confiscated drug products [7]. Young people, people with mental health problems, homeless people, drug users (injectors), and men who have sex with men are particularly vulnerable to NPSs [2].

### 3.2. Common Characteristics of NPSs

NPSs include mainly chemical derivates, chemical analogues (with one or few chemical modifications added in such a way as to alter the structure of psychoactive substances) or mimetics (which are chemically different, but still compounds that are designed to mimic, or that claim to mimic the effects of banned, illicit traditional street drugs) [8,9,10]. Based on their psychotropic effects, NPSs can be classified as stimulants, hallucinogens or empathogens/entactogens [11]. According to the UNODC classification, substances classified as NPSs include: synthetic cannabinoids, synthetic cathinones, phenethylamines, piperazines, ketamine, plant-based psychoactive substances (e.g., kratom (Mitragyna speciosa), khat (Catha edulis)), and other substances, including tryptamines, aminoindanes, phencyclidinetype substances [12], synthetic opioids, prescribed medications; performance and image-enhancing drugs [10,12].

NPSs are usually resorted to for reasons similar to those that drive the use of traditional drugs; in addition, NPSs are more easily available, at lower prices, are usually legal and cannot be detected in mandatory drug screening (involving prisoners, drug drivers, users in the army and public safety occupations, etc.) [2].

NPSs are commercialized as ‘legal highs’ or ‘smart drugs’, and are advertised as ‘safer’, ‘legal’ alternatives to illicit or controlled drugs [9]. Suppliers advertise NPSs with effective marketing strategies and with the aim of circumventing drug abuse legislation. NPSs are often labelled as ‘not for human consumption’ [1,2,12].

The NPS market is widely known to be dynamic, continuously and rapidly changing. Some NPSs emerged quickly and then disappeared (since 2013 this has happened with around 60 NPS), while others are continuously used among a small group of users (around 80 NPS, e.g., several synthetic cannabinoids, mephedrone and derivatives, several amphetamine analogues) [2]. The main reason for there being «always new NPS» is due to the response of various authorities to legal definitions and laws that likewise change continuously. Consequently, NPS suppliers are creating new chemical variations offering new alternatives to products that have become restricted, so attempting to circumvent drug controls by offering new products that could become legal (with the objective of ‘staying legal’), as substances differ from a chemical structure that has been deemed illegal. Despite the increasing number of NPSs appearing in drug markets, the overall size of the market remains relatively small when compared with other classic illicit drug markets [1,2,12].

NPSs are mainly manufactured in chemical laboratories in East and South-East Asia (the major sources and original locations of NPSs detected and/or used in other regions) and are then legally imported (as chemicals or as packaged products e.g., over the Internet by companies based mainly in China) into Europe, the US and other regions [1,2,12]. NPSs are produced without mandatory standards or controls, while no list of active pharmacological agents and no safety information are provided [1,2]. Most NPSs have little or even no history of medical use [2].

### 3.3. Illicit Opioids, NSOs

According to the US Food and Drug Administration (FDA) the illicit market for opioids includes internationally controlled substances (e.g., heroin) and prescription medicines that could be diverted from the legal market or produced as counterfeit medicines (fake products manufactured illegally in clandestine laboratories), which may contain fentanyl and fentanyl analogues [13,14]. The pills and powders containing fentanyl and analogues pose a threat to public health because of the variable quantity and potency of the active components (e.g., fentanyl may be 50–100 times more potent than morphine, carfentanil may be 10,000 times more potent than morphine). Such products may prove particularly dangerous when sold in the street to unsuspecting customers, especially opioid naive subjects [2].

According to the U.S. Drug Enforcement Administration (DEA) “fentanyl-related substances include any substance not otherwise controlled in any schedule (i.e., not included under any other Administration Controlled Substance Code Number) that is structurally related to fentanyl by one or more of the following modifications: replacement of the phenyl portion of the phenethyl group by any monocycle, whether or not further substituted in or on the monocycle; substitution in or on the phenethyl group with alkyl, alkenyl, alkoxyl, hydroxyl, halo, haloalkyl, amino or nitro groups; substitution in or on the piperidine ring with alkyl, alkenyl, alkoxyl, ester, ether, hydroxyl, halo, haloalkyl, amino or nitro groups; replacement of the aniline ring with any aromatic monocycle whether or not further substituted in or on the aromatic monocycle; and/or replacement of the N-propionyl group by another acyl group” [15].

NSOs include various analogues of fentanyl and newly emerging non-fentanyl compounds. NSO are used as stand-alone products, adulterants in heroin or constituents of counterfeit prescription medications [7].

According to UNODC, starting in 2014 NSOs appearing on the recreational drug market in the form of fentanyl mainly derived from the clandestine manufacturing (rather than from the diversion of prescribed pharmaceutical products containing fentanyl) of novel fentanyl analogues and other synthetic opioids that have never been approved for medical use [2]. Between 2012 and 2016 fourteen fentanyl analogues and three synthetic opioids belonging to other structural groups were reported to the UNODC by countries in East Asia, Europe and North America [1,2,12]; in the last five years twelve additional synthetic opioids have entered the illicit opioid market [2]. The increased availability of potent synthetic opioids, the non-medical use of synthetic opioids, the use of heroin (introduction of illicitly manufactured fentanyl into the heroin market), use of illicit pills and powder containing NSOs (fentanyl and analogues) by unsuspecting customers. Many users are, in fact, unknowingly consuming these compounds as adulterants in products sold as heroin, or as counterfeit pain killers, so leading to the recent increase in overdose deaths, especially in North America [16,17,18], and, similarly, in Canada [19] or in Miami, where an increase of 600% in fentanyl-related deaths was reported from 2014 to 2015, with an additional 200% from 2015 to 2016) [20]. According to the DEA, the current fentanyl crisis in the United States is largely fueled by illicitly manufactured fentanyl and its analogues, which are either illegally imported as such or synthesized from imported precursors [2,21].

NSOs include fentanyl and its analogues (e.g., acetylfentanyl, butyrfentanyl, furanylfentanyl, ocfentanil; fentanyl analogues developed between the 1960s and 1990s), novel fentanyl analogues such as acrylfentanyl and *para*-fluoroisobutyrfentanyl and new synthetic opioids such as AH-7921 (a benzoamide), MT-45 (a piperazine) and U-47700 (a compound closely related to AH-7921) [2].

Table 1, Table 2 and Table 3 present the common characteristics of novel opioids (designer, non-pharmaceutical fentanyls and other forms) as NPSs: most famous brand (street) names, the forms in which they appear on the market, means of use, neurobiology, intentionality of use. For all NSOs fatalities have been reported.

### 3.4. Kratom and O-Desmethyltramadol

According to UNODC [2], until 2013 the only NSOs that had been reported were kratom and O-desmethyltramadol.

#### 3.4.1. Kratom—Mitragyna Speciosa

This herbal product (mitragynine, obtained from the plant Mitragyna speciose) contains two active opioid compounds, whose International Union of Pure and Applied Chemistry (IUPAC) names are methyl (*E*)-2-[(2*S*,3*S*,12b*S*)-3-ethyl-8-methoxy-1,2,3,4,6,7,12,12b-octahydroindolo[2,3-a]-quinolizin-2-yl]-3-methoxyprop-2-enoate) and methyl (*E*)-2-[(2*S*,3*S*,7a*S*,12b*S*)-3-ethyl-7a-hydroxy-8-methoxy-2,3,4,6,7,12b-hexahydro-1*H*-indolo[2,3-a]quinolizin-2-yl]-3-methoxyprop-2-enoate (or 7-hydroxymitragynine) [22]. Mitragynine acts primarily via opioid receptors (receptor agonists) and is one third as potent as morphine and three times as potent as codeine [23,24]. 7-Hydroxymitragynine has potent mu and kappa receptor selectivity [23,24], with opioid receptor affinity up to 17 times that of morphine [23,24,25]; while it is present in the plant in much smaller quantities than mitragynine, it is a major contributing factor for the analgesic properties of the plant (due to its being a more potent opioid agonist). Mitragynine-pseudoindoxyl (a major component of kratom that has aged or been stored for extended periods), a mitragynine oxidation product, acts as a fairly selective mu-opioid agonist with little affinity for receptors [22].

The short-term effects of kratom mimic those of standard opioid agonists: nausea, dizziness, itching, constipation, sexual dysfunction [24,26]. According to Prozialeck [27] and other authors [27,28,29,30,31], the most common effects are anxiety, irritability, nausea, and vomiting. Due to high doses, or the use of concentrated extracts, the serious toxic reactions reportedly associated with kratom were tachycardia, seizures and liver damage [32,33,34,35,36]. Adverse effects due to the chronic use of kratom are sleep and eating disorders, dry mouth, facial hyperpigmentation, polyuria, psychosis, addiction [24,37], acute respiratory distress syndrome [38], hypothyroidism [39], intrahepatic cholestasis or toxic hepatitis [33,34], cardiotoxicity or dysrhythmia [34], seizure or coma [34,40,41]. Fatalities have usually been reported as a result of concomitant polydrug use [24,42,43,44,45,46,47,48], while other serious effects arising from the use of alleged kratom products have been reported [28,45,46,49,50], for instance, adulteration involving a product known as krypton, which was touted as a highly potent form of kKratom but contained high amounts of O-desmethyltramadol [51]. Withdrawal symptoms consisting of sweating, anxiety, restlessness, tremor and cravings for the substance; for these, a reducing regime of dihydrocodeine and lofexidine proved to be effective [52].

#### 3.4.2. O-Desmethyltramadol

O-Desmethyltramadol (IUPAC name: 3-[(1*R*,2*R*)-2-[(dimethylamino)methyl]-1-hydroxycyclo-hexyl]phenol), also known as O-demethyl tramadol, O-demethyltramadol, O-demethyltramadol hydrochloride or O-DT, is an opioid analgesic and the main active metabolite of tramadol [22]. This metabolite is considerably more potent as a mu opioid agonist and is believed to have 2–4 times the analgesic efficacy of the parent compound. O-DT itself is not sold as a prescription treatment or over the counter [22].

### 3.5. Fentanyl, Other Non-Pharmaceutical Fentanyls and Illicitly Manufactured Fentanyl Analogues

According to UNODC, NSOs were reported from North America, Latin America, Canada, Asia, Europe, Africa and elsewhere, and include illicitly manufactured fentanyl, analogues of fentanyl and various non-fentanyl compounds [2].

#### 3.5.1. Fentanyl

Fentanyl (IUPAC name: *N*-phenyl-*N*-[1-(2-phenylethyl)piperidin-4-yl]propanamide) is a synthetic, lipophilic phenylpiperidine opioid agonist with analgesic and anesthetic properties [22]. Fentanyl was developed for pharmaceutical use in 1960 by Paul Janssen in Belgium, and introduced in 1970 in the United States. It is a leading analgesic and anesthetic agent due to its 50–100 times higher potency than that of morphine, its shorter onset, and quicker absorption by the human body [22,53]. Fentanyl is primarily a mu-opioid agonist that selectively binds to the mu-receptor in the central nervous system. The drug is used in the medical management of severe chronic pain and postoperative pain, as well as in general and regional anesthesia [22,54]. Pharmaceutical fentanyl is also diverted for abuse [7,22], while illicitly manufactured fentanyl represents an additional threat to public health [2,22,55] Fentanyl can be absorbed into the body via inhalation, oral exposure or ingestion, or skin contact. Absorption through the skin may contribute to systemic toxicity. It is not known whether fentanyl can be absorbed systemically through the eye. Fentanyl can be administered intravenously, intramuscularly or as a skin patch (transdermally) [22]. According to O’Donnell et al. in 2016, in ten states in the US illicitly manufactured fentanyl and fentanyl analogues were a key factor driving a fivefold increase in opioid overdose deaths; 700 deaths tested positive for fentanyl analogs (most frequently, those analogues were carfentanil, furanylfentanyl, acetylfentanyl) [56].

Like fentanyl those fentanyls approved for medical use and others fentanyl analogues are internationally controlled under the 1961 Single Convention on Narcotic Drugs, acetylfentanyl from 2016 [54]. The three fentanyl analogues with legitimate human medical use are remifentanil, alfentanil, and sufentanil. Currently sufentanil, approximately 10–20 times less potent (500 to 1000 times the efficacy of morphine per weight) than carfentanil, is the maximum strength fentanyl analogue registered for use in humans [22].

#### 3.5.2. Carfentanyl

Carfentanyl or carfentanil (IUPAC name: methyl 1-(2-phenylethyl)-4-(*N*-propanoylanilino)- piperidine-4-carboxylate) is an analogue of fentanyl, and is one of the most potent opioids known and used commercially [22]. Carfentanyl was first synthesized in 1974 by a team of chemists at Janssen Pharmaceutical which included Paul Janssen and marked under the brand name Wildnil [22,54]. It has been approved for use in veterinary medicine as a general anaesthetic agent or as a tranquillizing agent for large-animal use only, as its extreme potency makes it inappropriate for use in humans. It has a quantitative potency approximately 10,000 times that of morphine and 100 times that of fentanyl [7,22,54], with activity in humans starting at about 1 microgram. Carfentanyl is a very potent agonist of opioid receptors, acting primarily on the mu (some kappa and delta) opioid receptors [22].

Carfentanyl is laced with or disguised as heroin, thus leading to hundreds of opioid overdoses, many of them fatal [56,57,58,59,60,61,62]. It is the most potent commercially available opioid in the world. Despite all the dangers it poses, is not currently under international control [2].

#### 3.5.3. Acetylfentanyl

Acetylfentanyl (IUPAC name: *N*-phenyl-*N*-[1-(2-phenylethyl)piperidin-4-yl]acetamide) or acetyl fentanyl [22], also known as *N*-(1-phenethylpiperidin-4-yl)-*N*-phenylacetamide, des-methylfentanyl acetyl fentanyl, MCV 4848, NIH 10485) has never been marketed for medical or veterinary use [7,63,64,65,66,67,68,69,70,71,72,73,74]. Acetylfentanyl exhibits a pharmacological profile similar to that of fentanyl and other opioid analgesic compounds [73]. Acetylfentanyl is 5 to 15 times more potent than heroin, 16 times more potent than morphine, while its potency is one third that of fentanyl 7 [7,73] and it is 15 times less active than fentanyl [73,75]. In addition, the range between the effective dose (ED_50_) and the lethal dose (LD_50_) of acetylfentanyl is narrower than that of morphine and fentanyl, which increases the risk of a fatal overdose [73]. Thus, its abuse is likely to pose quantitatively greater risks to public health and safety than abuse of traditional opioid analgesics such as morphine [73]. Acetylfentanyl has been appeared on illicit markets in America, Europe, Japan, China, and Australia in the last few years [76]. It is used by opioid-dependent individuals illicitly (sometimes being called the “First Apostle of Extinction”) as a substitute for fentanyl (acting as its controlled precursor) or for heroin [77].

Typically seized by the police on drug markets as powder, tablets, capsules, blotters, or as liquids and in injectable formulations [2,75,76,78,79]. It can typically be administered orally, nasally (using sprays), by snorting, smoking or by intravenous injection [75,77,79]. Case reports of intoxication and deaths involving acetylfentanyl have been disclosed from the United States, the United Kingdom, Sweden, Japan, Russia, Germany, Poland, and other European countries, while, in parallel, seizures have dramatically increased in the US, some European Countries, China, and Japan since 2015 [1,2,12,14,64,67,76]. Since 2016 it has come under international control [2,73].

The abuse of opioid analgesics has resulted in large numbers of drug treatment admissions, emergency department visits, and fatal overdoses, as a result of which intravenous routes of administration and histories of drug abuse have been documented [73]. Acetylfentanyl has been associated with numerous fatalities (e.g., 39 overdose deaths due to acetylfentanyl abuse in 2013 and again in 2014 in US) [73].

#### 3.5.4. Butyrylfentanyl

Butyrylfentanyl (IUPAC name: *N*-phenyl-*N*-[1-(2-phenylethyl)piperidin-4-yl]butanamide) or butyr-fentanyl or butyryl-fentanyl or *N*-(1-phenylethyl)-4-piperidinyl)-*N*-phenylbutyramide or butyrfentanyl; butyl fentanyl; BF) [7,22,80,81], is a potent short-acting synthetic opioid analgesic, an analogue of fentanyl with around one quarter of its potency [22]; it is about seven times more potent than morphine [82]. It is an agonist at mu opioid receptors. Butyrylfentanyl has no current legitimate clinical applications [22]. Butyrylfentanyl can be obtained through illicit sources, and there is often no information on its purity and potency, thus posing significant adverse health risks to its users [83]. It is seized in the form of powder, tablets, capsules, blotters, liquids or in injectable formulas [75,76,77,78,79,84]. It could be administered orally, nasally (using sprays), by snorting, smoking or by intravenous injection [75,77,78,79]. Fatal cases have been reported, with reference to at least 40 confirmed fatalities associated with the misuse and/or abuse of butyrylfentanyl [80,81]. It is under international control [2].

#### 3.5.5. 4-Fluorobutyrylfentanyl

4-Fluorobutyrylfentanyl (IUPAC name: *N*-(4-fluorophenyl)-*N*-[1-(2-phenylethyl)piperidin-4-yl]butanamide) or 4-fluorobutyrfentanyl or *para*-fluoroisobutyryl fentanyl [22]. or 4-fluoro-butyr-fentanyl or 4-FBF [10,80,81]. It is usually presented in the form of powder or a nasal spray [61], liquids, tablets, capsules [75,78,79], mostly used orally, nasally (with sprays), by snorting, smoking, by intravenous injection [75,77,79], rectally or by heating the drug and inhaling the vapor. It is available on the Internet added to heroin, often without the user’s knowledge [7]. Fatalities have been reported [20,54,85].

Other NPS fentanyl analogues reported to the UNODC in the period 2012–2016 are [2]:

- Furanylfentanyl (IUPAC name: *N*-phenyl-*N*-[1-(2-phenylethyl)piperidin-4-yl]furan-2-carboxamide;hydrochloride) or FU-F HCl or furafentanyl or Fu-F [7,22,44,70,86];
- Ocfentanil (IUPAC name: *N*-(2-fluorophenyl)-2-methoxy-*N*-[1-(2-phenylethyl)piperidin-4-yl]acetamide [22]. It has short-acting opioid-like effects, the same potency as fentanyl, and can be injected, snorted or smoked [6,87,88];
- Acryloylfentanyl (IUPAC name: *N*-phenyl-*N*-[1-(2-phenylethyl)piperidin-4-yl]prop-2-enamide) or acrylfent or acrylfentanyl, acryloyl-F, Acr-F, ACF, [18F]ACF [2,22,75,89,90,91]. Acrylfentanyl is used in medicine as an adjunct to general anesthesia during surgery and for pain management [92]. Present on drug market in liquid and tablet form, less frequently in powder or capsule form [4,91], and is taken nasally (as a nasal solution or by snorting), orally or by intravenous injection [4,75,91,93];
- α-Methylfentanyl (IUPAC Name: *N*-[1-(α-methyl-β-phenyl)ethyl-4-piperidyl]propionanilide) was sold under street names such as “China white,” “China girl,” “Persian white,” “egg white,” “crocodile,” “synthetic heroin” and is 7000 times more potent than morphine [54,77];
- 4-Fluorofentanyl (IUPAC name: *N*-(4-fluorophenyl)-*N*-[1-(2-phenylethyl)piperidin-4-yl]propanamide or *p*-fluorofentanyl or parafluorofentanyl or [22]. *p*-Fluorofentanyl was developed by Janssen Pharmaceutica in the 1960s, and in the early 1980s was sold for a short period on the US black market [94];
- Others: valerylfentanyl (IUPAC name: *N*-phenyl-*N*-[1-(2-phenylethyl)piperidin-4-yl]-pentanamide [7,22].; 4-methoxybutyrfentanyl (IUPAC name: *N*-(4-methoxyphenyl)-*N*-[1-(2-phenylethyl)piperidin-4-yl]butanamide) or 4-methoxybutyrylfentanyl or 4-MeO-BF [22].; depropionylfentanyl (IUPAC name: *N*-phenyl-1-(2-phenylethyl)piperidin-4-amine or despropionylfentanyl or ANPP [22].; isobutyrfentanyl (IUPAC name: 2-methyl-*N*-phenyl-*N*-[1-(2-phenylethyl)piperidin-4-yl]propenamide or isobutyryl fentanyl [22].; despropionyl-2-fluorofentanyl, (iso)butyr-F-fentanyl *N*-benzyl analogue, methoxyacetylfentanyl, *para*-fluoroisobutyrfentanyl, etrahydrofuranylfentanyl, *para*-fluoroisobutyrfentanyl and others.

### 3.6. New Generation of Synthetic Opioids: Structurally Atypical Synthetic Opioids

Since 2010 NSOs with chemical structures different from fentanyl appeared on the recreational drug market, e.g., AH-7921 (a benzoamide), MT-45 (a piperazine) [2,54]. AH-7921 (IUPAC name: 3,4-dichloro-*N*-[[1-(dimethylamino)cyclohexyl]methyl]benzamide) but it can also be found as 1-(3,4-dichlorobenzamidomethyl)cyclohexyldimethylamine, 3,4-dichloro-*N*-(1-(dimethylamino)cyclo- hexyl)methylbenzamide, or AH 7921 hydrochloride [7,22,49,95,96,97], known also as doxylam [10], or doxylan [95,98]. It is 0.8 times as potent as morphine [98,99]. On the drug market it is sold as a free base and as a hydrochloride salt in a white/off-white powder form [10,75,98,100,101]. The usual way of introduction is orally, by smoking, by nasal insufflation, sublingual application, and less often by intravenous injection [98], by a combination of insufflation and oral consumption, or rectal administration (in the powder, tablet, capsule at doses ranging from 10 to 150 mg [102]. Adverse effects include feelings of depression, mild insomnia after withdrawal, seizures, hypertension, tachycardia [98,102] meiosis, hypothermia, sedation, respiratory depression, and inhibition of gastrointestinal propulsion, and death [95].

U-47700 (IUPAC name: 3,4-dichloro-*N*-[2-(dimethylamino)cyclohexyl]-*N*-methylbenzamide) or 3,4-dichloro-*N*-[2-(dimethylamino)cyclohexyl]-*N*-methylbenzamide HCl, “pink”, or “U4’’ is 7.5 times more potent than morphine; it appears in powders, tablets, and liquids [7,22,70,83,103,104,105,106,107]. Typical routes of administration are oral, nasal, intrarectal, by smoking, intravenous injection or by combinations of these routes [106,108]. A patient who survived an overdose presented with decreased mental status and decreased respiratory rate suggestive of an opioid toxidrome. Patients also commonly had tachycardia [59,109]. U-47700 has been associated with numerous cases of overdoses and overdose deaths [107,110].

MT-45 (IUPAC name: 1-cyclohexyl-4-(1,2-diphenylethyl)piperazine, is a mu/delta/sigma opioid receptor agonist, 3.5 times more potent than morphine [7,10,22,49,80,101,111]. Starting in 2013, MT-45 began appearing on the internet for sale as a ‘legal’ opioid [112]. MT-45 is present in herbal and chemical mixtures containing synthetic cannabinoids and/or synthetic cathinones (e.g., “Wow”) [10,112]. MT-45 is a piperazine derivative and is structurally unrelated to most other opioids. There are two enantiomers of MT-45 (R and S), both bound to opioid receptors, however (*S*)-(+)-MT-45 binds with a greater affinity than that of (*R*)-(−)-MT-45. In functional studies, (*S*)-(+)-MT-45 has an analgesic effect similar to that of morphine. In comparison, the analgesic effect of (*R*)-(−)-MT-45 is low. MT-45 has been demonstrated to produce physical dependence in mice. There are no published studies on the safety of MT-45 for human use [112]. Typical routes of administration include oral, nasal insufflation, snorting (intolerable level of irritation in some users), inhalation, intravenous and intramuscular injection, with varying reported doses; intrarectal use has also been reported [113,114]. Reported adverse effects include hair depigmentation and loss, folliculitis, dermatitis, painful intertriginous dermatitis, dry eyes, elevated liver enzymes, transverse white Mees’ lines (leukon y-chia striata) on the fingernails and toenails, disorganized keratinization, bilateral secondary cataracts requiring surgery [115,116], hearing loss and unconsciousness [115]. Deaths associated with MT-45 abuse have occurred in the US and in Europe; there have been at least 13 non-fatal overdoses associated with abuse of MT-45 [112].

### 3.7. Other Reported NSOs with Chemical Structures Different from Fentanyl

W-18 (IUPAC name: (*NE*)-4-chloro-*N*-[1-[2-(4-nitrophenyl)ethyl]piperidin-2-ylidene]benzenesulfonamide or 4-chloro-*N*-(1-(4-nitrophenethyl)piperidin-2-ylidene)benzenesulfonamide [22], has an analgesic potency 10,000 times greater than morphine [80,117]. W-15 (IUPAC name: (*NE*)-4-chloro-*N*-[1-(2-phenylethyl)piperidin-2-ylidene]benzenesulfonamide [22,117]. IC-26 (IUPAC name: 4-ethylsulfonyl-*N*,*N*-dimethyl-4,4-diphenylbutan-2-amine;hydrochloride) or “methiodone” (a methadone analogue) or methiodone hydrochloride [10,22]. U-50488 (IUPAC name: 2-(3,4-dichlorophenyl)-*N*-methyl-*N*-[(1*R*,2*R*)-2-pyrrolidin-1-ylcyclohexyl]acetamide [22,70]. U-49900 (IUPAC name: 3,4-dichloro-*N*-(2-(diethylamino)cyclohexyl)-*N*-methylbenzamide [99]. nortilidine (IUPAC name: ethyl (1*R*,2*S*)-2-(methylamino)-1-phenylcyclohex-3-ene-1-carboxylate) is an NMDA receptor antagonist and dopamine reuptake inhibitor equipotent to morphine 10 and U-51754 [99].

### 3.8. Potential Risks for First Responders and Reducing the Risk of NSO Exposure

The best-known example of a fentanyl analogues-related threat to public safety dates back to 2002, when Russian Special Forces used a chemical aerosol probably consisting of a mixture of carfentanil and remifentanil against Chechen terrorists to rescue hostages in the Dubrovka theatre. Due to the combination of aerosol use, inadequate medical care (with only a narrow margin of safety between therapeutic and lethal doses in humans, and a high chance of a lethal outcome in the absence of prompt and appropriate medical intervention), the operation resulted in 125 deaths [118].

The National Institute for Occupational Safety and Health (NIOSH) suggests strategies to reduce the risk of inadvertent exposure to NSOs with a potential risk of overdose and death among first responders (e.g., clandestine laboratories) [119]: «avoid testing drugs in the field; instead, transport to a laboratory: use respiratory protection when handling or testing fentanyl or other illicit substances; use gloves when handling any unidentifiable drug; wear eye protection to minimize risk of eye and mucus membrane exposure; consider wearing coveralls, boot covers, and protective sleeves; avoid airborne dispersal of substance during a sweep, and avoid opening/closing suspicious bags or containers; carry an adequate supply of naloxone to use in the case of accidental exposure».

The American College of Medical Toxicology and American Academy of Clinical Toxicology analyzed the risk of exposure to illicit synthetic opioids, and concluded that [119]: “inhalation is less of a concern because airborne concentrations are unlikely to reach threatening levels; it is unlikely that limited skin exposure to tablets or powder would cause significant opioid toxicity, and if toxicity were to occur it would not develop rapidly, allowing time for removal”; mucous membrane exposure can be prevented by using OSHA-rated splash protection.

## 4. Discussion

Young people, people with mental health problems, homeless people, decompensated or not-in-treatment drug users (injectors), and men who have sex with men are particularly vulnerable to NSOs misuse. These substances include mainly chemical derivates, chemical analogues (with one or few chemical modifications to alter their structure) or mimetics (which are chemically different, but still compounds that are designed to mimic, or that claim to simulate the effects of banned, illicit traditional street drugs). All these kind of compounds can increase the risk of overdose, and therefore it is necessary to reduce this risk with acute and long-term interventions.

Opiate overdose can be recognized based on specific signs and symptoms such as pinpoint pupils and respiratory depression, while mydriasis is a sign of opiate abstinence. Patients with suspected opioid overdose should be treated if the respiratory rate is <12 breaths per minute or if their oxygen saturation is less than 90%). The initial treatment of hypoxic patients, in the Emergency Room (ER), is to provide oxygen and assisted ventilation as necessary. In general, this procedure involves the release of airways and the application of ventilation with a breathing bag and a mask for the delivery of oxygen. In the ER but also in the streets, generally, opioid overdose is reversed by the intravenous use of naloxone. Naloxone is a non-selective, short-acting opioid receptor antagonist with established success for the treatment of overdose of short-acting opiates, such as heroin, and the overdose of prescription opiates, but can also control NSO overdoses [120].

Treating opioid dependence is the best way to reduce the risk of opioid overdose. The primary pharmacological approach to managing opioid dependence involves agonist opioid maintenance treatment (AOT) [121,122]. AOT implies the substitution of the illegal drug by a prescribed opioid which has good μ-receptor activity and longer half-life [123] and aims to avoid, or at least reduce, euphoric symptoms, drug craving and withdrawal symptoms [122,123]. The medications most frequently used are methadone and buprenorphine [122]. Generally speaking, it is possible to treat Opioid Use Disorder (OUD) according to two principal different methodologies: Harm Reduction Treatment (HRT) and AOT.

During HRT, medication dosage and duration of treatment are usually limited, regardless of clinical indication, which suggests the value of increased dosage or treatment duration [124]. Patients are allowed to negotiate the lowering of dosages regardless of urinalyses and to have their medication tapered earlier than advisable by the scientific literature [125,126].

On the opposite side, AOT is focused on pharmacological maintenance. Four are the phases of AOT. During the first (induction phase) patients are safety transferred from street opioid to an opioid agonist. During the stabilization phase, the medication doses are gradually increased to blocking doses until the point is reached where the patient is tolerant to street opioid. Once this requirement is fulfilled, the patient is defined as having been “stabilized”. No upper limit for dosage exists. The dosage is increased to reflect the results of urinalyses. Patients are not allowed to raise or lower the dose by themselves. During maintenance phase patients remain on a consistent agonist dose level that will enable him/her to function properly. During the fourth phase (medically supervised withdrawal) gradual reduction of agonist dose is applied until the patient is completely drug-free; typically, in association with psychosocial support [122,127,128,129]. AOT is more efficient in treating OUD than HRT [130], and the blocking doses are highly protective against overdoses [131].

Naloxone is well known to be an antidote to opiate overdose, which could be defined as a potentially lethal acute intoxication by mu-opiate agonists [132,133,134]. Overdosing among people exposed to opiates may take one of at least two forms. On one hand, accidental overdosing resulting from the excessive administration of opiate drugs for various purposes (mainly sedation or for treating painful syndromes), or interactions which increase the expected peak level of opiate agonism or amplify its expected effect at a pharmacodynamic level. On the other hand, overdosing is one possible consequence of a symptomatic behavior found in opiate addiction—that is, blindness to risk in the self-administration of opiates by addicted individuals, whether alone or in combination with other substances. Furthermore, other cases may be due to intentional self-poisoning, in other words, suicidal behavior. That epidemiological distinction also has a pathophysiological meaning that is linked to differences in behavioral style, where the dynamics of administration may lead to accidental overdosing and where there are environmental circumstances in which overdoses can be expected [135,136].

Obviously, opioid antagonists have no impact on the incidence or recurrence of overdosing, or the average severity of overdose accidents as they are experienced by patients. We assume that the new interest in developing a better formulation of an antidote to overdosing has arisen in the trail of an increasing concern about overdose epidemics, partly sustained by the presence of new NSOs. A reasonable response to overdose epidemics should certainly be based on overdose prevention, rather than antidote optimization, considering that adequate means of prevention are so easy to implement. Stated as a paradox, antidote optimization may be completely neutralized by a treatment strategy that fails to focus on long-term treatment, and thus favors overdose epidemics (in addition to the failure to counteract them), while also inducing an unfavorable change in overdosing typology. In other words, one should avoid letting the disease worsen, because that kind of evolution always paves the way for more severe relapses, before anyone becomes concerned about the optimized management of acute complications.

In the thinking of an addictionologist, the issue of overdose prevention is closely connected with the wider issues of addiction treatment and harm reduction [125], and looms as highly dependent on the effectiveness of treatments to put off cravings and reduce impulsive drug-using behavior. Correct treatment planning should therefore first implement secondary prevention (i.e., addiction treatment) to pave the way for any emergency treatment. The average severity of overdosing can, in fact, be reduced by means of anticraving treatment to be spread through the addicted population [127,137,138,139,140,141,142,143]. Not only would patients be protected against overdosing arising from a state of high-level tolerance to opiates (from implementing a narcotic blockade), but they would also be less likely to display further risk factors for lethal overdosing (e.g., comorbidity or polyabuse) [144,145,146]. It is known that the behavioral stabilization of patients while on standard treatment with agonists turns out to be effective in preventing complications such as impaired global health status, infective diseases, and polyabuse [147,148,149,150].

On the other hand, procedures which lead patients from a condition of acquired tolerance to a condition of low or no tolerance at all actually increase the risk of overdosing, due to a condition of treatment-free relapsing disorder, which addiction is known to be [151]. Reviewing data about overdose risk factors, authors agree in stating that major risk factors include being out of any treatment, and having no tolerance to opiates [152,153,154]. Thus, the most protective way, whether in a rehabilitation or a harm reduction perspective, is to be in methadone or buprenorphine treatment. Ineffective dosages may be enough to prevent overdoses, while reducing their lethal nature by being able to rely on the favorable ratio low dosages create between tolerance and craving. Treatment regimens that tend to provide a blockade in the absence of a direct anticraving effect have a less favorable profile, since they combine no or low tolerance with unchanged, or only marginally diminished craving. The introduction of newly formulated opioid antagonists to be taken intranasally [155] certainly increases the manageability of an antidote to overdosing, by avoiding the intravenous administration route [156,157,158,159,160]. Its role should, however, be reasonably conceived within a global healthcare plan whose main aim is to spread and optimize agonist treatment coverage and quality by using an adequate dose over the long term. Otherwise, the impact of overdose management as a life-saving option would have little impact on the destiny of opiate-addicted people [161].

When treatments are not administered by applying the above-mentioned correct standards, some features will turn up to impair what is actually the lifesaving effectiveness of opioid antagonists. First of all, the incidence of overdoses will increase, but with a relatively higher increase in the percentage of cases which take place among older addicts, who are prematurely discharged from treatment, and are unjustifiably assessed as presumably healed, whereas they are certainly ill. Such overdoses are quite likely to take place unexpectedly, and find the person alone, having enough money to supply him- or herself with a high dose of a potent, good-quality opiate (due to intermittent and apparently successful rehab), and—what counts most—no tolerance. Thereby, the risk estimation resulting from the ratio between craving intensity and tolerance is even more unfavorable than it is for street addicts [146]. Data show that overdosing has become relatively more common among older addicts, who may run the further risk of a greater level of somatic morbidity. Moreover, treatment leaks do mean that the same individuals are put at risk of overdose recurrence, for at least two reasons: recurrent detoxifications, and distrust felt towards treatment facilities, as long as these patients believe they have relapsed ‘in spite of’ what were, in reality, successfully accomplished programs. The distrust that may be felt towards potentially effective (long-term) programs as a result of badly performed short-term programs is a vicious circle which brings about an increased incidence of between-treatment overdose accidents.

Thus, near-death prevention of overdoses would be good news as long as addicts found themselves in a suitable condition to receive prompt administration of the antidote. Street overdosing, or overdosing in the context of group drug-taking is the target condition for intranasal naloxone, since it is a quicker and easier to administer solution, so making up for the lack of instantaneous bioavailability by its time-saving administration procedure. Even so, saving the lives of addicted people remains a strategic issue, since lethal accidents and the morbidity of addicted people are both rooted in the core symptoms and relapsing course of their disease. As a result, any lack of concern, or, worse, the refusal to accept the need to head towards long-term agonist-assisted recovery and relapse prevention, will hamper healing perspectives such as the effectiveness of life-saving opioid antagonists. When and where addicts are overdosing, opioid antagonists need to be available on the spot, which is hardly likely if overdosing trends are changing towards the higher frequency of solitary, unexpected cases, and situations where older patients are suffering from more than one form of comorbidity. Everyone would certainly agree that resuscitating more patients is a meaningful goal to achieve, unless that aim puts those same patients at a higher risk of being in repeated need of resuscitation, through a series of short-term treatments, with resuscitation coming earlier than if it were only due to the disease itself. Likewise, one would certainly agree that we have only a few minutes to prevent death from overdose, whereas overdose prevention can be attempted every day by performing correct agonist treatment and discouraging premature treatment dropout or withdrawal. The incidence of overdosing and its lethal nature are indicators of something being wrong, either with the treatment design or with its management, since ongoing standard treatment is known to be largely and promptly effective in preventing overdosing. Overdose epidemics can be interpreted as a finger pointing unmistakably towards the need to improve treatment standards at a very basic level.

## 5. Conclusions

Illicit fentanyl, its analogues and other NSOs pose an alarming risk to public health and life, with high abuse potential and severe adverse effects. NSOs are used as heroin substitutes or as adulterants in illicit street drugs—a fact which users are mostly unaware of. Illicit fentanyl and its analogues set up unpredictable, potentially unsafe conditions that are a threat to first responders and others. In NSO toxicity (opioid toxidrome), due to high mu-opioid affinity, larger naloxone doses are required than those of a classic heroin overdose [80,114]. As long as addicts are dying from overdose or similar causes, there is something more constructive to do than waiting for addicts to overdose on heroin at a place located near a remedy, as if to say, within reach of naloxone, so it may be hoped that when data about overdoses indicate a need for prevention, health care professionals will be more constructive than simply staying focused on the prospects of resuscitation, considering that the need for resuscitation could have been avoided well before that predicament was reached.

## Figures and Tables

**Table 1:** Common characteristics of novel synthetic opioids NSOs: most famous brand (street) names, forms in which they appear on the market, means of use, neurobiology, and intentionality of use.

Typology	Most Famous Brand Names	Forms in Which Found on the Market
Non-medical fentanyl, illicitly manufactured fentanyl	“China White”, “Synthetic Heroin”, “China Girl”, “Chinatown”, “Tango & Cash”, “TNT”, “Drop Dead”, “Flatline”, “Lethal Injection”, “Poison”, “Apache”, “Dance Fever”, “Great Bear”, “Perc-o-Pops”, “Lollipops”.	Tablets: buccal (Fentora™) and sublingual (Abstral^®^); oral transmucosal lozenges (Actiq^®^), film Onsolis^®^, spray: sublingual (Subsys™) and nasal (Lazanda^®^); transdermal patches (Ionsys^®^, (Duragesic^®^ and generics; brand names: Duragesic, Duragesic Mat, Ionsys, Fentanyl Transdermal System Novaplus), injectable formulations (Sublimaze^®^).
Kratom (Mitragyna speciosa)	“Thom”, “Thang”, “Biak”, “Krathom”, “Kakuam”, “Biak-Biak”, “Ketum”, “Mambog”, “Natural Kratom leaf”, “Phoriatm Borneo white vein”, “Phoriatm green”, “Phoriatm maeng da kava”, “Phoriatm Borneo green vein”, “Kratom shot” (liquid formulation), “Green vein extra strength” (liquid formulation) “Super Premium Powder”, “three ‘80X Extract”, Super Concentrated Liquid”, “‘Bali Kratom”, “Indo Kratom”, “Kratom tincture”, “Kratom Resin”, “Kratom Regular”.	Naturally occurring kratom leaf and marketed kratom supplements: crushed or powdered dried leaves, powder, kratom preparations fortified with extracts from other leaves, extracts and resin, gum, tinctures, capsules filled with powdered kratom, tablets, liquid formulation.
O-Desmethyltramadol	O-Desmethyltramadol	Liquid form; kratom (leaves of Mitragyna speciosa) could also contain o-desmethyltramadol.
Novel Fentanyl derivatives	Usually added to or substituted for heroin, often without the user’s knowledge; e.g., acetylfentanyl, butyrylfentanyl, furanyl fentanyl, 4methyl fentanyl and other forms; usually not approved for medical use	Powders (usually mixed with heroin or other illicit drugs), tablets (counterfeit prescriptions pills), nasal sprays, liquids.
New generation of Novel Synthetic Opioids, structurally atypical synthetic opioids	Usually added to or substituted for heroin, often without the user’s knowledge; e.g., U-51754, U-47700, AH-7921, MT-45 and others; usually not approved for medical use.	Powders (usually mixed with heroin or other illicit drugs), tablets (counterfeit prescriptions pills), nasal sprays, liquids.

**Table 2:** Common characteristics of NSOs: means of use, neurobiology, and intentionality of use.

Typology	Means of Use	Neurobiology
Non-medical fentanyl, illicitly manufactured fentanyl	Transdermal fentanyl patches: smoked (placed in glass containers and heated or fentanyl scratched) or taken intranasally (fentanyl powder snorted); parenterally or orally (gel contents removed from the patches, oral ingestion of lozenges); parenteral (patches simmered in a water and injected intravenously, intramuscularly); frozen patches cut into pieces and then chewed, placed under the tongue, or in the cheek cavity for drug absorption through the oral mucosa or inserted into the rectum.	Binds to mu-receptor but also to kappa and delta-type opioid receptors.
Kratom (Mitragyna speciosa)	Fresh or dried leaves chewed or brewed into tea, ice-cold cocktails from kratom leaves, dried leaves smoked	-Mitragynine produces opioid-like effects predominantly via mu- and delta-opioid receptor agonism; Mitragynine-pseudoindoxyl (oxidation product of mitragynine) acts as a fairly selective opioid agonist with little affinity for receptors; 7-Hydroxymitragynine is a much more potent opioid agonist, with potent mu and kappa receptor selectivity.
O-Desmethyltramadol	Same means as for kratom.	Mu-opioid receptor activation
Novel Fentanyl derivatives	Orally, sublingual application, nasally—by smoking or by nasal insufflation, intrarectally, intravenous injection, intramuscular injection or by combinations of these routes.	Acts primarily on the mu (plus some kappa and some delta) opioid receptors.
New generation of Novel Synthetic Opioids, structurally atypical synthetic opioids	Orally, sublingual application, nasally—by smoking or by nasal insufflation, intrarectally, intravenous injection, intramuscular injection or by combinations of these routes.	Mu/delta/sigma opioid receptor agonist.

**Table 3:** Common characteristics of NSOs: intentionality of use.

Typology	Intentionality of Use
Non-medical fentanyl, illicitly manufactured fentanyl	Yes
Kratom (Mitragyna speciosa)	Yes, but e.g., up to 500% artificially elevated concentrations of 7-hydroxymitragynine exceeded that found in naturally occurring material or o-desmethyltramadol added
O-Desmethyltramadol	Main active metabolite of tramadol, but not sold as a prescription treatment or over the counter. Kratom (leaves of Mitragyna speciosa; most famous form of krypton) could also contain o-desmethyltramadol.
Novel Fentanyl derivative	Not for fentanyl analogues—usually added to heroin or other illicit drugs, often without the user’s knowledge.
New generation of Novel Synthetic Opioids, structurally atypical synthetic opioids	Not for NSOs—usually added to heroin or other illicit drugs, often without the user’s knowledge.
